# Crystal structure analysis of ethyl 3-(4-chloro­phen­yl)-1,6-dimethyl-4-methyl­sulfanyl-1*H*-pyrazolo[3,4-*b*]pyridine-5-carboxyl­ate

**DOI:** 10.1107/S2056989020002479

**Published:** 2020-02-25

**Authors:** H. Surya Prakash Rao, Ramalingam Gunasundari, Jayaraman Muthukumaran

**Affiliations:** a Department of Chemistry and Biochemistry, School of Basic Sciences and Research, Sharda University, Greater Noida 201306, India; b Department of Chemistry, Pondicherry University, Puducherry 605014, India; c Department of Biotechnology, School of Engineering and Technology, Sharda University, Greater Noida 201306, India

**Keywords:** crystal structure, pyrazolo­pyridine, biological activity

## Abstract

In the title compound, C_18_H_18_ClN_3_O_2_S, the dihedral angle between the fused pyrazole and pyridine rings is 3.81 (9)°. The benzene ring forms dihedral angles of 35.08 (10) and 36.26 (9)° with the pyrazole and pyridine rings, respectively. In the crystal, weak C—H⋯O hydrogen bonds connect mol­ecules along [100].

## Abstract 2

In the title compound, the dihedral angle between the fused pyrazole and pyridine rings is 3.81 (9)°. The benzene ring forms dihedral angles of 35.08 (10) and 36.26 (9)° with the pyrazole and pyridine rings, respectively. In the crystal, weak C—H⋯O hydrogen bonds connect mol­ecules along [100].

## Chemical context   

The nitro­gen-containing heterocyclic motif is a component in many medicinally important drugs. Mol­ecules built around the pyrazolo­pyridine core structure exhibit diverse medicinal properties that include anti-microbial, anti-viral, anti-fungal, anti-hypertensive, analgesic, anti-cancer, anti-inflammatory, anti-Alzheimer’s, anti-diabetic, anti-nociceptive, anti-tuberculosis, and anti-leishmanial activities (Hardy, 1984 ▸; Hawas *et al.* 2019 ▸; de Mello *et al.* 2004 ▸; Panchal *et al.* 2019 ▸; El-Gohary *et al.* 2019 ▸). In addition, some pyrazolo­pyridines have found uses for the treatment of hemorrhagic stress, infertility, and drug addiction (Parmar *et al.* 1974 ▸). Specifically, they act as inhibitors of enzymes such as glycogen synthase kinase-3 (Witherington *et al.* 2003 ▸) and as inhibitors for adenosine receptors (Timóteo *et al.* 2008 ▸). Furthermore, they have been identified as promising inhibitors of cycline dependent kinase, xanthine oxidase, inter­leukin-6 (IL-6), tumor necrosis factor alpha (TNF-α), phospho­diesterase-4, NAD(P)H oxidases and cholesterol formation (Gökhan-Kelekçi *et al.* 2007 ▸; Panchal *et al.* 2019 ▸; Fathy *et al.* 2015 ▸). Considering the aforementioned importance of derivatives of pyrazolo­pyridine, we have carried out a single-crystal X-ray diffraction study on the title compound and have analyzed the structure in terms of geometrical parameters, conformation, and inter­mol­ecular hydrogen-bonding inter­actions.
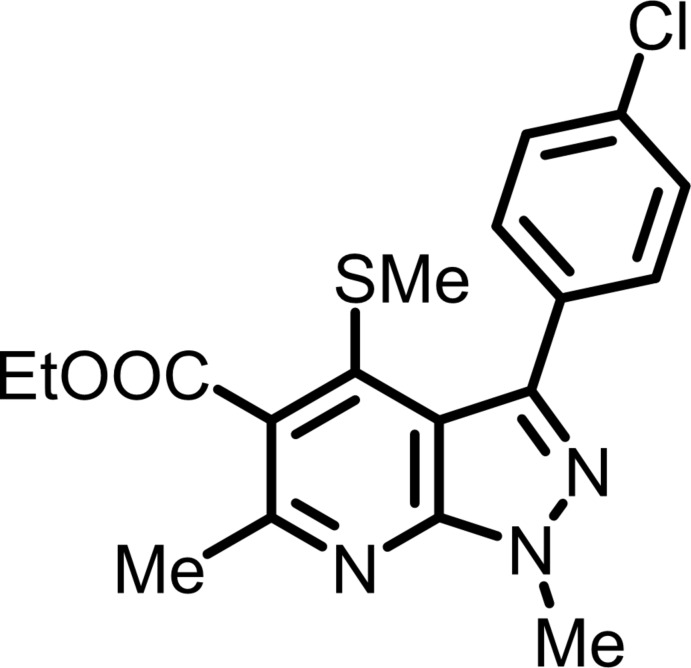


## Structural commentary   

**Figure 1:**
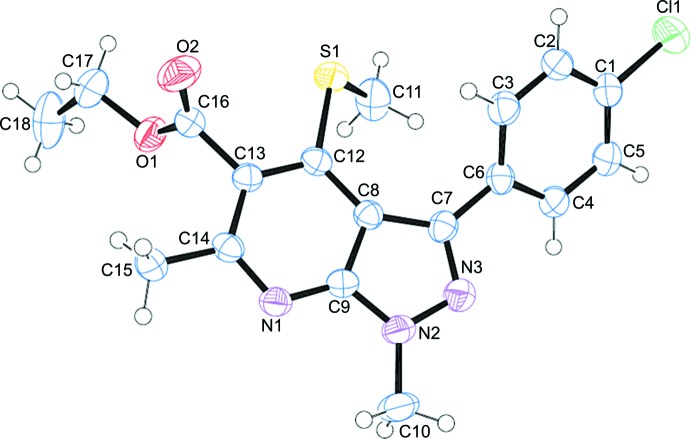
The mol­ecular structure of the title compound with the atom-numbering scheme and displacement ellipsoids drawn at the 50% probability level

The title compound has pyrazole­[3,4-*b*]pyridine motif that is decorated by several substituents shown in Fig. 1 ▸. The chloro­phenyl (C_6_H_4_Cl) group attached to the pyrazolo­pyridine moiety exhibits an (−)anti­clinal conformation [N3—C7—C6—C3 = −141.96 (19)°], as does the methyl­thio (SCH_3_) group attached to the pyrazolo­pyridine unit [C11—S1—C12—C13 = −128.93 (15) °] while the –COOC_2_H_5_ group attached to the pyrazolo­pyridine moiety has an (+)anti-periplanar conformation [N1—C14—C13—C16 = 177.00 (15)°, as do the methyl group attached to the pyridine sub-structure [C9—N1—C14—C15 = −176.20 (16)°] and the methyl group attached to the pyrazole ring (NCH_3_) [C10—N2—C9—C8: −178.42 (19)°]. The fused pyrazole and pyridine rings are not exactly planar, subtending a dihedral angle of 3.81 (9)°. The dihedral angle between the planes of the benzene and pyrazole rings is 35.08 (10)° and that between the benzene and pyridine rings is 36.26 (9)°.

## Supra­molecular features   

**Figure 2:**
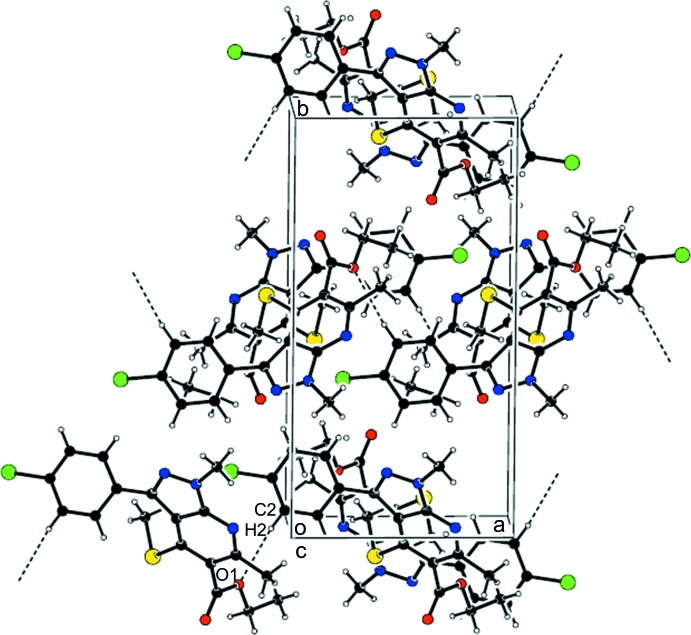
The crystal packing of title compound, viewed along the *c* axis, showing the weak inter­molecular C—H⋯O hydrogen bonds as dotted lines

In the crystal, weak C—H⋯O hydrogen bonds link mol­ecules into chains along [100] (Table 1 ▸ and Fig. 2 ▸).

## Database survey   

A search for the pyrazolo­pyridine scaffold in the Cambridge Structural Database (CSD, Version 5.40; Groom *et al.*, 2016 ▸) gave 236 hits. Of these, the structures most closely related to the title compound are FIZLEI (ethyl 2,7-di­amino-3,4-di­cyano-5-phenyl­pyrazolo­[1,5-*a*]pyridine-6-carboxyl­ate; Naik *et al.* 2019 ▸), ALAFID (Wu *et al.* 2016 ▸), DAWKAQ {[2-(4-chloro­phen­yl)pyrazolo­[1,5-*a*]pyridin-3-yl(phen­yl)methanone; Ravi *et al.* 2017 ▸}, NADPIU [3-(4-chloro­phen­yl)pyrazolo­[1,5-*a*]pyridine; Wu *et al.* 2016 ▸] and ZOJWAW (Barrett *et al.* 1996 ▸). The geometrical parameters of the –COOCH_2_CH_3_ substituent in the title compound are comparable with those reported for FIZLEI. Similarly, the geometrical parameters of the –C_6_H_4_Cl unit in the title compound are comparable with those for in DAWKAQ and NADPIU. The bond lengths of the pyrazolo­[3,4-*b*]pyridine scaffold of the title compound are closer to those in NADPIU. The pyrazolo­pyridine moiety (N1–N3/C7–C9/C12–C14) of the title compound is approximately plan, as is also observed for FIZLEI, ALAFID, DAWKAQ, NADPIU and ZOJWAW. Apart from the CSD database, two other important databases, namely Drug Bank (database for FDA-approved drugs, drugs under investigation or in clinical trials, *etc*; Law *et al.* 2013 ▸) and ZINC (database for commercially available compounds; Irwin *et al.* 2005 ▸) were also surveyed. The former database is used for drug repurposing or drug re-profiling studies, and latter for high-throughput virtual screening against the binding site of drug target proteins to identify promising and putative inhibitors. In the Drug Bank database, there were 31 hits, based on a 0.5 similarity threshold, whereas the ZINC search gave only three hits (ZINCIDs: ZINC45166781, ZINC3852638 and ZINC39053824). Out of 31 mol­ecules identified in the Drug Bank database, two mol­ecules were in the approved drug category namely riciguat (accession No: DB08931, similarity score: 0.55) and teletristat ethyl (accession No: DB12095, similarity score: 0.511). The remaining 29 mol­ecules belong to the experimental, investigational or other categories.

## Synthesis and crystallization:   

To a solution of 3-(4-chloro­phen­yl)-1-methyl-1*H*-pyrazol-5-amine (125 mg, 0.65 mmol) and ethyl 2-(bis­(meth­ylthio)­meth­ylene)-3-oxo­butano­ate (145 mg, 0.65 mmol) in toluene (5 ml) under a blanket of dry N_2_, a catalytic amount of tri­fluoro­acetic acid (TFA; 30 mol%) was added. The resulting mixture was refluxed for 12 h, while monitoring progress by TLC (hexa­ne:ethyl acetate, 99:1). After completion of the reaction, the resulting mixture was subjected to purification by column chromatography to furnish 182 mg of the title compound in 75% yield as a colourless solid, m.p. 415.85 K, *R*_f_ = 0.3 (hexa­ne:ethyl acetate 99:01). A sample suitable for single-crystal X-ray analysis was obtained by recrystallization from dry methanol.

## Refinement   

Crystal data, data collection and structure refinement details are summarized in Table 2 ▸. Hydrogen atoms were placed in calculated positions, with C—H = 0.93–0.97 Å and refined using a riding model with *U*_iso_(H) = 1.2 *U*_eq_(C) or 1.5 *U*_eq_(C-meth­yl).

## Supplementary Material

Crystal structure: contains datablock(s) I. DOI: 10.1107/S2056989020002479/lh5946sup1.cif

Structure factors: contains datablock(s) I. DOI: 10.1107/S2056989020002479/lh5946Isup2.hkl

Supporting information file. DOI: 10.1107/S2056989020002479/lh5946Isup3.cml

CCDC reference: 1977404

Additional supporting information:  crystallographic information; 3D view; checkCIF report

## Figures and Tables

**Table 1:** Hydrogen-bond geometry (Å, °)

*D*—H⋯*A*	*D*—H	H⋯*A*	*D*⋯*A*	*D*—H⋯*A*
C2—H2⋯O1^i^	0.93	2.59	3.513 (2)	170

Symmetry code: (i) 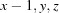.

**Table 2:** Experimental details

Crystal data	
Chemical formula	C_18_H_18_ClN_3_O_2_S
*M*_r_	375.86
Crystal system, space group	Monoclinic, *P*2_1_/*a*
Temperature (K)	298
*a*, *b*, *c* (Å)	8.9995 (5), 16.7778 (11), 12.3595 (8)
β (°)	98.892 (6)
*V* (Å^3^)	1843.8 (2)
*Z*	4
Radiation type	Mo *K*α
μ (mm^−1^)	0.34
Crystal size (mm)	0.65 × 0.6 × 0.24

Data collection	
Diffractometer	Agilent Xcalibur Eos
Absorption correction	Multi-scan (*CrysAlis PRO*; Agilent, 2014 ▸)
*T*_min_, *T*_max_	0.857, 1.000
No. of measured, independent and observed [*I* > 2σ(*I*)] reflections	13882, 4340, 3323
*R*_int_	0.027
(sin θ/λ)_max_ (Å^−1^)	0.682

Refinement	
*R*[*F* ^2^ > 2σ(*F* ^2^)], *wR*(*F* ^2^), *S*	0.049, 0.161, 1.10
No. of reflections	4340
No. of parameters	230
H-atom treatment	H-atom parameters constrained
Δρ_max_, Δρ_min_ (e Å^−3^)	0.30, −0.41

Computer programs: *CrysAlis PRO* (Agilent, 2014 ▸), *SHELXT* (Sheldrick, 2015*a* ▸), *SHELXL2018* (Sheldrick, 2015*b* ▸), *ORTEP-3 for Windows* (Farrugia, 2012 ▸), *PLATON* (Spek, 2020 ▸) and *Mercury* (Macrae *et al.*, 2020 ▸).

## supplementary crystallographic information

### Crystal data

**Table:**

C_18_H_18_ClN_3_O_2_S	*F*(000) = 784
*M**_r_* = 375.86	*D*_x_ = 1.354 Mg m^−^^3^
Monoclinic, *P*2_1_/*a*	Mo *K*α radiation, λ = 0.71073 Å
*a* = 8.9995 (5) Å	Cell parameters from 4472 reflections
*b* = 16.7778 (11) Å	θ = 3.9–29.0°
*c* = 12.3595 (8) Å	µ = 0.34 mm^−^^1^
β = 98.892 (6)°	*T* = 298 K
*V* = 1843.8 (2) Å^3^	Block, colourless
*Z* = 4	0.65 × 0.6 × 0.24 mm

### Data collection

**Table:**

Agilent Xcalibur Eos diffractometer	4340 independent reflections
Radiation source: Enhance (Mo) X-ray Source	3323 reflections with *I* > 2σ(*I*)
Detector resolution: 15.9821 pixels mm^-1^	*R*_int_ = 0.027
ω scans	θ_max_ = 29.0°, θ_min_ = 3.9°
Absorption correction: multi-scan (CrysAlis PRO; Agilent, 2014)	*h* = −11→12
*T*_min_ = 0.857, *T*_max_ = 1.000	*k* = −22→22
13882 measured reflections	*l* = −15→16

### Refinement

**Table:**

Refinement on *F*^2^	Primary atom site location: structure-invariant direct methods
Least-squares matrix: full	Secondary atom site location: difference Fourier map
*R*[*F*^2^ > 2σ(*F*^2^)] = 0.049	Hydrogen site location: inferred from neighbouring sites
*wR*(*F*^2^) = 0.161	H-atom parameters constrained
*S* = 1.10	*w* = 1/[σ^2^(*F*_o_^2^) + (0.1*P*)^2^] where *P* = (*F*_o_^2^ + 2*F*_c_^2^)/3
4340 reflections	(Δ/σ)_max_ = 0.006
230 parameters	Δρ_max_ = 0.30 e Å^−^^3^
0 restraints	Δρ_min_ = −0.41 e Å^−^^3^

### Special details

**Table:**

Geometry. All esds (except the esd in the dihedral angle between two l.s. planes) are estimated using the full covariance matrix. The cell esds are taken into account individually in the estimation of esds in distances, angles and torsion angles; correlations between esds in cell parameters are only used when they are defined by crystal symmetry. An approximate (isotropic) treatment of cell esds is used for estimating esds involving l.s. planes.

### Fractional atomic coordinates and isotropic or equivalent isotropic displacement parameters (Å^2^)

**Table:**

	*x*	*y*	*z*	*U*_iso_*/*U*_eq_	
S1	0.38706 (5)	−0.06089 (3)	0.65888 (4)	0.04572 (19)	
Cl1	−0.26383 (7)	0.13979 (4)	0.63284 (7)	0.0751 (3)	
O1	0.77428 (16)	−0.12709 (8)	0.67467 (12)	0.0460 (4)	
N1	0.73909 (16)	0.02099 (9)	0.95211 (12)	0.0363 (4)	
N2	0.57052 (18)	0.12776 (9)	0.97183 (14)	0.0404 (4)	
O2	0.62392 (19)	−0.20707 (9)	0.75585 (14)	0.0599 (4)	
N3	0.42863 (18)	0.15124 (10)	0.92994 (14)	0.0409 (4)	
C9	0.6093 (2)	0.06010 (10)	0.92252 (14)	0.0335 (4)	
C7	0.37469 (19)	0.09887 (11)	0.85219 (15)	0.0347 (4)	
C14	0.75435 (19)	−0.04529 (11)	0.89531 (15)	0.0340 (4)	
C3	0.1239 (2)	0.04255 (12)	0.77078 (17)	0.0421 (5)	
H3	0.161730	−0.008533	0.785668	0.051*	
C8	0.48642 (19)	0.03902 (11)	0.84112 (14)	0.0323 (4)	
C15	0.8936 (2)	−0.09402 (12)	0.93020 (17)	0.0430 (5)	
H15A	0.968939	−0.061386	0.972304	0.064*	
H15B	0.930807	−0.113697	0.866557	0.064*	
H15C	0.869892	−0.138103	0.974026	0.064*	
C6	0.21737 (19)	0.10825 (11)	0.79948 (15)	0.0345 (4)	
C12	0.51150 (19)	−0.02733 (11)	0.77555 (14)	0.0329 (4)	
C2	−0.0231 (2)	0.05183 (12)	0.72086 (17)	0.0435 (5)	
H2	−0.083292	0.007504	0.701057	0.052*	
C5	0.0072 (2)	0.19448 (12)	0.73163 (18)	0.0463 (5)	
H5	−0.032853	0.245383	0.719599	0.056*	
C13	0.64421 (19)	−0.06941 (10)	0.80566 (14)	0.0322 (4)	
C4	0.1552 (2)	0.18420 (12)	0.78090 (17)	0.0410 (4)	
H4	0.214097	0.228738	0.801982	0.049*	
C10	0.6609 (3)	0.17282 (13)	1.05805 (19)	0.0525 (5)	
H10A	0.723443	0.137017	1.105641	0.079*	
H10B	0.596079	0.201357	1.099350	0.079*	
H10C	0.722908	0.209945	1.026288	0.079*	
C16	0.6758 (2)	−0.14269 (11)	0.74373 (15)	0.0372 (4)	
C1	−0.0800 (2)	0.12802 (13)	0.70063 (17)	0.0439 (5)	
C11	0.3529 (3)	0.02945 (16)	0.57990 (18)	0.0586 (6)	
H11A	0.303494	0.067547	0.620089	0.088*	
H11B	0.290191	0.017846	0.511611	0.088*	
H11C	0.446915	0.051040	0.565940	0.088*	
C17	0.8220 (3)	−0.19386 (15)	0.6139 (2)	0.0620 (7)	
H17A	0.741429	−0.209688	0.556464	0.074*	
H17B	0.847277	−0.238963	0.662333	0.074*	
C18	0.9549 (3)	−0.16898 (19)	0.5657 (2)	0.0784 (9)	
H18A	0.928634	−0.124659	0.517341	0.118*	
H18B	0.988342	−0.212571	0.525243	0.118*	
H18C	1.034070	−0.153518	0.623018	0.118*	

### Atomic displacement parameters (Å^2^)

**Table:**

	*U*^11^	*U*^22^	*U*^33^	*U*^12^	*U*^13^	*U*^23^
S1	0.0408 (3)	0.0500 (3)	0.0424 (3)	0.0014 (2)	−0.0061 (2)	−0.0113 (2)
Cl1	0.0410 (3)	0.0734 (5)	0.1034 (6)	0.0093 (3)	−0.0123 (3)	−0.0214 (4)
O1	0.0491 (8)	0.0400 (8)	0.0522 (8)	0.0015 (6)	0.0179 (7)	−0.0078 (6)
N1	0.0350 (8)	0.0336 (8)	0.0387 (8)	−0.0032 (6)	0.0005 (6)	−0.0019 (7)
N2	0.0426 (9)	0.0337 (9)	0.0426 (9)	−0.0007 (6)	−0.0009 (7)	−0.0074 (7)
O2	0.0696 (10)	0.0392 (9)	0.0749 (11)	−0.0140 (7)	0.0236 (8)	−0.0115 (8)
N3	0.0411 (9)	0.0371 (9)	0.0436 (9)	0.0035 (7)	0.0040 (7)	−0.0016 (7)
C9	0.0356 (9)	0.0319 (9)	0.0327 (9)	−0.0041 (7)	0.0042 (7)	−0.0007 (7)
C7	0.0375 (9)	0.0305 (9)	0.0368 (9)	0.0014 (7)	0.0084 (7)	0.0017 (7)
C14	0.0308 (8)	0.0332 (9)	0.0370 (9)	−0.0045 (7)	0.0027 (7)	0.0027 (8)
C3	0.0426 (10)	0.0332 (10)	0.0522 (12)	0.0002 (8)	0.0122 (9)	0.0019 (9)
C8	0.0312 (8)	0.0317 (9)	0.0339 (9)	−0.0015 (7)	0.0048 (7)	0.0008 (7)
C15	0.0360 (9)	0.0422 (11)	0.0476 (11)	0.0032 (8)	−0.0030 (8)	−0.0006 (9)
C6	0.0323 (8)	0.0373 (10)	0.0357 (9)	0.0010 (7)	0.0105 (7)	0.0015 (8)
C12	0.0306 (8)	0.0361 (9)	0.0319 (9)	−0.0044 (7)	0.0049 (7)	0.0006 (7)
C2	0.0375 (10)	0.0416 (11)	0.0531 (12)	−0.0053 (8)	0.0122 (8)	−0.0061 (9)
C5	0.0408 (10)	0.0391 (11)	0.0590 (13)	0.0088 (8)	0.0075 (9)	−0.0040 (10)
C13	0.0320 (8)	0.0312 (9)	0.0334 (9)	−0.0030 (7)	0.0050 (7)	−0.0004 (7)
C4	0.0376 (9)	0.0355 (10)	0.0511 (11)	−0.0009 (8)	0.0101 (8)	−0.0049 (9)
C10	0.0624 (13)	0.0420 (12)	0.0490 (12)	−0.0044 (10)	−0.0044 (10)	−0.0149 (10)
C16	0.0333 (9)	0.0364 (10)	0.0405 (10)	0.0003 (7)	0.0015 (7)	−0.0031 (8)
C1	0.0349 (10)	0.0494 (12)	0.0487 (11)	0.0037 (8)	0.0098 (8)	−0.0067 (9)
C11	0.0595 (13)	0.0778 (17)	0.0367 (10)	0.0189 (12)	0.0022 (9)	0.0032 (11)
C17	0.0615 (14)	0.0577 (15)	0.0692 (15)	0.0088 (11)	0.0179 (12)	−0.0218 (12)
C18	0.0790 (19)	0.092 (2)	0.0719 (17)	0.0399 (16)	0.0366 (15)	0.0135 (16)

### Geometric parameters (Å, º)

**Table:**

S1—C12	1.7752 (18)	C15—H15C	0.9600
S1—C11	1.803 (3)	C6—C4	1.396 (3)
Cl1—C1	1.746 (2)	C12—C13	1.388 (2)
O1—C16	1.347 (2)	C2—C1	1.385 (3)
O1—C17	1.450 (2)	C2—H2	0.9300
N1—C14	1.333 (2)	C5—C1	1.383 (3)
N1—C9	1.340 (2)	C5—C4	1.387 (3)
N2—C9	1.359 (2)	C5—H5	0.9300
N2—N3	1.360 (2)	C13—C16	1.499 (2)
N2—C10	1.449 (2)	C4—H4	0.9300
O2—C16	1.195 (2)	C10—H10A	0.9600
N3—C7	1.337 (2)	C10—H10B	0.9600
C9—C8	1.420 (2)	C10—H10C	0.9600
C7—C8	1.442 (2)	C11—H11A	0.9600
C7—C6	1.473 (2)	C11—H11B	0.9600
C14—C13	1.426 (2)	C11—H11C	0.9600
C14—C15	1.502 (3)	C17—C18	1.476 (3)
C3—C2	1.380 (3)	C17—H17A	0.9700
C3—C6	1.399 (3)	C17—H17B	0.9700
C3—H3	0.9300	C18—H18A	0.9600
C8—C12	1.415 (2)	C18—H18B	0.9600
C15—H15A	0.9600	C18—H18C	0.9600
C15—H15B	0.9600		
			
C12—S1—C11	101.91 (10)	C1—C5—H5	120.4
C16—O1—C17	117.03 (16)	C4—C5—H5	120.4
C14—N1—C9	114.90 (15)	C12—C13—C14	122.00 (17)
C9—N2—N3	111.25 (15)	C12—C13—C16	120.27 (16)
C9—N2—C10	127.70 (17)	C14—C13—C16	117.74 (16)
N3—N2—C10	121.05 (16)	C5—C4—C6	121.27 (18)
C7—N3—N2	107.38 (15)	C5—C4—H4	119.4
N1—C9—N2	124.05 (17)	C6—C4—H4	119.4
N1—C9—C8	128.45 (16)	N2—C10—H10A	109.5
N2—C9—C8	107.43 (16)	N2—C10—H10B	109.5
N3—C7—C8	110.17 (16)	H10A—C10—H10B	109.5
N3—C7—C6	117.73 (15)	N2—C10—H10C	109.5
C8—C7—C6	131.99 (16)	H10A—C10—H10C	109.5
N1—C14—C13	121.99 (16)	H10B—C10—H10C	109.5
N1—C14—C15	116.87 (16)	O2—C16—O1	124.26 (18)
C13—C14—C15	121.14 (17)	O2—C16—C13	124.64 (17)
C2—C3—C6	121.48 (19)	O1—C16—C13	111.07 (15)
C2—C3—H3	119.3	C5—C1—C2	121.04 (19)
C6—C3—H3	119.3	C5—C1—Cl1	119.79 (16)
C12—C8—C9	115.20 (15)	C2—C1—Cl1	119.16 (16)
C12—C8—C7	140.99 (16)	S1—C11—H11A	109.5
C9—C8—C7	103.74 (15)	S1—C11—H11B	109.5
C14—C15—H15A	109.5	H11A—C11—H11B	109.5
C14—C15—H15B	109.5	S1—C11—H11C	109.5
H15A—C15—H15B	109.5	H11A—C11—H11C	109.5
C14—C15—H15C	109.5	H11B—C11—H11C	109.5
H15A—C15—H15C	109.5	O1—C17—C18	108.3 (2)
H15B—C15—H15C	109.5	O1—C17—H17A	110.0
C4—C6—C3	117.87 (17)	C18—C17—H17A	110.0
C4—C6—C7	120.25 (17)	O1—C17—H17B	110.0
C3—C6—C7	121.83 (17)	C18—C17—H17B	110.0
C13—C12—C8	116.96 (16)	H17A—C17—H17B	108.4
C13—C12—S1	117.71 (14)	C17—C18—H18A	109.5
C8—C12—S1	125.32 (13)	C17—C18—H18B	109.5
C3—C2—C1	119.14 (19)	H18A—C18—H18B	109.5
C3—C2—H2	120.4	C17—C18—H18C	109.5
C1—C2—H2	120.4	H18A—C18—H18C	109.5
C1—C5—C4	119.13 (19)	H18B—C18—H18C	109.5
			
C9—N2—N3—C7	−0.4 (2)	C9—C8—C12—S1	−173.01 (13)
C10—N2—N3—C7	179.58 (18)	C7—C8—C12—S1	3.2 (3)
C14—N1—C9—N2	178.40 (16)	C11—S1—C12—C13	−128.93 (15)
C14—N1—C9—C8	1.6 (3)	C11—S1—C12—C8	51.29 (17)
N3—N2—C9—N1	−175.78 (16)	C6—C3—C2—C1	−1.2 (3)
C10—N2—C9—N1	4.2 (3)	C8—C12—C13—C14	−2.9 (3)
N3—N2—C9—C8	1.6 (2)	S1—C12—C13—C14	177.35 (13)
C10—N2—C9—C8	−178.42 (19)	C8—C12—C13—C16	177.08 (15)
N2—N3—C7—C8	−0.9 (2)	S1—C12—C13—C16	−2.7 (2)
N2—N3—C7—C6	175.72 (15)	N1—C14—C13—C12	−3.1 (3)
C9—N1—C14—C13	3.7 (2)	C15—C14—C13—C12	176.81 (16)
C9—N1—C14—C15	−176.20 (16)	N1—C14—C13—C16	177.00 (15)
N1—C9—C8—C12	−7.2 (3)	C15—C14—C13—C16	−3.1 (3)
N2—C9—C8—C12	175.55 (15)	C1—C5—C4—C6	0.1 (3)
N1—C9—C8—C7	175.22 (17)	C3—C6—C4—C5	−2.5 (3)
N2—C9—C8—C7	−2.00 (19)	C7—C6—C4—C5	−179.85 (17)
N3—C7—C8—C12	−174.7 (2)	C17—O1—C16—O2	−1.7 (3)
C6—C7—C8—C12	9.4 (4)	C17—O1—C16—C13	176.52 (18)
N3—C7—C8—C9	1.81 (19)	C12—C13—C16—O2	−79.4 (3)
C6—C7—C8—C9	−174.18 (18)	C14—C13—C16—O2	100.5 (2)
C2—C3—C6—C4	3.1 (3)	C12—C13—C16—O1	102.39 (19)
C2—C3—C6—C7	−179.64 (17)	C14—C13—C16—O1	−77.7 (2)
N3—C7—C6—C4	35.3 (2)	C4—C5—C1—C2	1.8 (3)
C8—C7—C6—C4	−148.98 (19)	C4—C5—C1—Cl1	−177.07 (15)
N3—C7—C6—C3	−141.96 (19)	C3—C2—C1—C5	−1.3 (3)
C8—C7—C6—C3	33.8 (3)	C3—C2—C1—Cl1	177.61 (15)
C9—C8—C12—C13	7.2 (2)	C16—O1—C17—C18	−166.05 (19)
C7—C8—C12—C13	−176.6 (2)		

### Hydrogen-bond geometry (Å, º)

**Table:**

*D*—H···*A*	*D*—H	H···*A*	*D*···*A*	*D*—H···*A*
C2—H2···O1^i^	0.93	2.59	3.513 (2)	170

Symmetry code: (i) *x*−1, *y*, *z*.
